# HER2/HER3 pathway in biliary tract malignancies; systematic review and meta-analysis: a potential therapeutic target?

**DOI:** 10.1007/s10555-016-9645-x

**Published:** 2016-12-15

**Authors:** Salvatore Galdy, Angela Lamarca, Mairéad G. McNamara, Richard A. Hubner, Chiara A. Cella, Nicola Fazio, Juan W. Valle

**Affiliations:** 1 Unit of Gastrointestinal Medical Oncology and Neuroendocrine Tumors, European Institute of Oncology, Milan, Italy; 2 Department of Medical Oncology, The Christie NHS Foundation Trust, Manchester, UK; 3 Institute of Cancer Sciences, Department of Medical Oncology, The Christie NHS Foundation Trust, University of Manchester, Wilmslow Road, Manchester, M20 4BX UK

**Keywords:** Biliary tract cancer, Cholangiocarcinoma, HER2 pathway, HER3 pathway, Systematic review, Meta-analysis

## Abstract

Human epidermal growth factor receptor 2 (HER2) overexpression and amplification have been reported as predictive markers for HER2-targeted therapy in breast and gastric cancer, whereas human epidermal growth factor receptor 3 (HER3) is emerging as a potential resistance factor. The aim of this study was to perform a systematic review and meta-analysis of the HER2 and HER3 overexpression and amplification in biliary tract cancers (BTCs). An electronic search of MEDLINE, American Society of Clinical Oncology (ASCO), European Society of Medical Oncology Congress (ESMO), and American Association for Cancer Research (AACR) was performed to identify studies reporting HER2 and/or HER3 membrane protein expression by immunohistochemistry (IHC) and/or gene amplification by *in situ* hybridization (ISH) in BTCs. Studies were classified as “high quality” (HQ) if IHC overexpression was defined as presence of moderate/strong staining or “low quality” (LQ) where “any” expression was considered positive. Of 440 studies screened, 40 met the inclusion criteria. Globally, HER2 expression rate was 26.5 % (95 % CI 18.9–34.1 %). When HQ studies were analyzed (*n* = 27 studies), extrahepatic BTCs showed a higher HER2 overexpression rate compared to intrahepatic cholangiocarcinoma: 19.9 % (95 % CI 12.8–27.1 %) vs. 4.8 % (95 % CI 0–14.5 %), respectively, *p* value 0.0049. HER2 amplification rate was higher in patients selected by HER2 overexpression compared to “unselected” patients: 57.6 % (95 % CI 16.2–99 %) vs. 17.9 % (95 % CI 0.1–35.4 %), respectively, *p* value 0.0072. HER3 overexpression (4/4 HQ studies) and amplification rates were 27.9 % (95 % CI 9.7–46.1 %) and 26.5 % (one study), respectively. Up to 20 % of extrahepatic BTCs appear to be HER2 overexpressed; of these, close to 60 % appear to be HER2 amplified, while HER3 is overexpressed or amplified in about 25 % of patients. Clinical relevance for targeted therapy should be tested in prospective clinical trials.

## Introduction

### Background

The prognosis for patients with advanced biliary tract cancers (BTCs) is very poor with a median overall survival of less than 12 months following treatment with systemic chemotherapy [1]. The term BTCs refers to a heterogeneous group of diseases encompassing cholangiocarcinoma (CC) [intrahepatic cholangiocarcinoma (IHCC), extrahepatic cholangiocarcinoma (EHCC)], gallbladder carcinoma (GBC), and ampulla of Vater carcinoma (AC). It is postulated that specific genetic and molecular aberrations vary between these subtypes and thus may provide predictive biomarkers of response to targeted therapy. Unfortunately, unlike other solid tumors, targetable biomarkers are lacking in BTCs and the cisplatin and gemcitabine combination remains gold standard first-line treatment worldwide in patients with advanced disease [2], with no proven benefit from targeted therapies as yet identified [3, 4]. Thus, biomarkers of response are urgently required in this challenging disease. The human epidermal growth factor receptor 2 (HER2), which belongs to the ErbB/(HER) family of receptor tyrosine kinases (TK), is a well-described predictive biomarker for anti-HER2 therapy in breast and gastric cancer [5, 6]. To date, previous clinical reports have suggested some activity of trastuzumab (an anti-HER2 monoclonal antibody) in association with chemotherapy in HER2 upregulated BTCs [7–10]. In contrast, trials exploring the role of anti-EGFR monoclonal antibodies and EGFR tyrosine kinase inhibitors (TKIs) have resulted in disappointing and/or conflicting findings [11–14]. This systematic review aims to quantify the reported HER2 and HER3 expression rates in BTC in order to provide useful data for the development of potential novel systemic-targeted strategies for use in future clinical trials.

### HER2/HER3 pathway

**Fig. 1:**
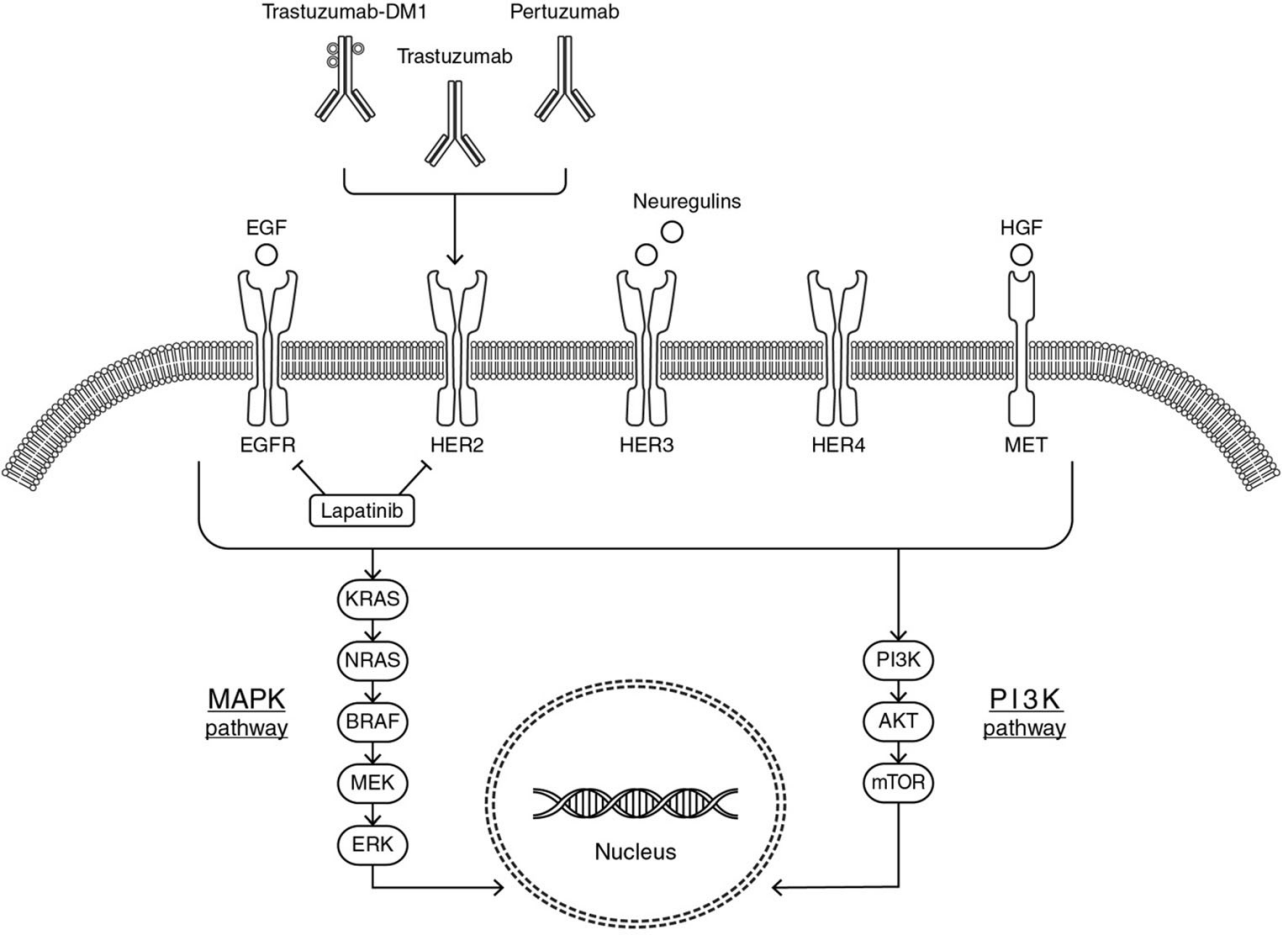
HER2/HER3 pathway and targeted therapy interaction

The HER family consists of four receptors [HER1 (EGFR), HER2, HER3, and HER4] with similar structure, consisting of four main parts: an extracellular ErbB ligand-binding domain, a single transmembrane lipophilic segment, an intracellular tyrosine kinase domain, and an intracellular C-terminal tail [15]. The extracellular ligand-binding region contains four domains (I–IV): domains I and III recognize and bind their corresponding ligands and domain II mediates receptor dimerization, whereas domain IV, interacting with domain II, leads to a negative feedback on the dimerization process [16]. Ligand binding to the extracellular domain results in receptor homo- or heterodimerization, a critical step in HER family-mediated signaling. Dimerization induces the activation of the intrinsic tyrosine kinase domain, by phosphorylation of specific tyrosine residues, leading to the activation of different downstream signaling cascades, including the mitogen-activated protein kinase (MAPK) proliferation pathway and phosphatidylinositol 3-kinase (PI3K)/protein kinase B (PKB or Akt) pro-survival pathway [17, 18] (Fig. 1).

HER2 and HER3 are known to have characteristics distinct from other HER family receptors. HER2 lacks a specific ligand, so it can form heterodimers only if it is trans-activated from other activated HER receptors (such as EGFR, HER3, and HER4) [19]. In addition to abnormal overexpression, HER2 is also able to spontaneously homodimerize [16]. In contrast to HER2, HER3 can bind multiple ligands (neuregulins) [20] but it lacks a functioning kinase domain [21] and is, therefore, unable to homodimerize and to induce downstream signaling pathway activation on its own. However, in the presence of HER3 ligands, HER3 may promote the kinase activity of EGFR or HER2 and thereby induce phosphorylation of the HER3 C-terminal tail inducing the PI3K/Akt pathway activation by creating heterodimers [20]. Although all four HER family receptors are capable of dimerizing with each other, HER2 is the preferred dimerization partner [19] and the HER2–HER3 dimer seems to be the most potent HER family dimer [22, 23]. Finally, HER3 has the ability to dimerize with both HER family members and non-HER family members such as mesenchymal epithelial transition (MET) receptor [24, 25], contributing to anti-HER2 therapy resistance. Thus, dysregulation of HER-mediated signaling pathways, through this complex mechanism, results in the growth and spread of cancer cells.

### HER2 and HER3 determination

The most commonly used methods to determine the HER2 and HER3 status, in formalin-fixed paraffin-embedded tissue, are (a) immunohistochemistry (IHC), which measures the number of HER2 and HER3 receptors on the cell surface and therefore detects receptor overexpression and (b) fluorescence or chromogenic *in situ* hybridization (FISH and CISH, respectively), which detects gene amplification by measuring the number of copies of the HER2 and HER3 gene in the nuclei of tumor cells.

Currently, standard clinical practice guidelines for HER2 status assessment are available for breast and gastric cancer only. In contrast, because HER3 status is not routinely analyzed, IHC and ISH techniques for assessing HER3 status have not been standardized.

The IHC and FISH scoring criteria are different for breast and gastric cancer [26, 27], reflecting intrinsic biological differences, including higher heterogeneity of HER2 membranous immunoreactivity in gastric cancer. In addition, in gastric cancer, different scales are used depending on the nature of the diagnostic specimen (surgical specimen vs. biopsy sample) [6, 27]; these criteria are summarized in Table 1.

**Table 1:** Standardized guidelines for HER2 analysis, adjusted from 2013 ASCO/CAP guidelines for the HercepTest™ scoring system in breast cancer [26] and standardized guidelines (for both surgical and biopsy specimen) for gastric adenocarcinoma [6, 27]

	0+ (negative)	1+ (weak; negative)	2+ (moderate; equivocal)	3+ (strong; positive)
HER2 expression (IHC)				
Breast cancer	No staining observed or membrane staining that is incomplete and is faint/barely perceptible and within ≤10 % of the invasive tumor cells	Incomplete membrane staining that is faint/barely perceptible and within >10 % of the invasive tumor cells	Circumferential membrane staining that is incomplete and/or weak/moderate and within >10 % of the invasive tumor cells or complete and circumferential membrane staining that is intense and within ≤10 % of the invasive tumor cells	Circumferential membrane staining that is complete and intense in >10 % of the cancerous cells
Gastric cancer; surgical specimens	No reactivity or membranous reactivity in <10 % of cells	Faint⁄barely perceptible membranous reactivity in >10 % of cells; cells are reactive only in part of their membrane	Weak to moderate complete or basolateral membranous reactivity in >10 % of tumor cells	Moderate to strong complete or basolateral membranous reactivity in >10 % of tumor cells
Gastric cancer; biopsy specimens	No reactivity or no membranous reactivity in any tumor cell	Faint/barely perceptible membranous reactivity irrespective of percentage of tumor cells	Weak to moderate complete, basolateral or lateral membranous reactivity irrespective of percentage of tumor cells	Strong complete, basolateral or lateral membranous reactivity irrespective of percentage of tumor cells
HER2 amplification (ISH)				
Breast cancer	HER2 FISH testing (gene copy number and HER2-to-CEP17 ratio) positive: HER2 gene copy number is greater than 6.0 (single probe) and in case of HER2 2+ if either HER2/CEP17 ratio is ≥2.0 regardless gene copy number or if HER2/CEP17 ratio is <2.0 with an average HER2 copy number ≥6.0 (dual probe)			
Gastric cancer	FISH amplified (positive): IHC/HER2 2+ tumor samples are considered FISH amplified if HER2/CEP17 ratio is ≥2			

*IHC* immunohistochemistry, *ISH in situ* hybridization

Data from published series of HER2 and HER3 expression varies both in terms of methodology, reporting, and subsequent utility. We therefore set out to undertake a systematic review (i.e., pooled analysis of HER2 and HER3 expression in published BTC series), to provide a “summary estimate” of such expression, with a view to informing the design of future clinical trials.

## Methods

### Study selection criteria

Eligible studies were those which met the following inclusion criteria: (1) studies reporting membrane expression by IHC and/or amplification by ISH of HER2 and/or HER3 data in human BTC tissue; (2) studies in which data for invasive/infiltrating tumors was available; and (3) original article publications (or abstracts, in the absence of a full publication); studies reporting preclinical data, reviews, and case reports were excluded. Studies in which data for the subgroup of patients with BTC was not available (i.e., when only combined results were reported including non-BTC primary disease sites such as hepatocellular carcinoma, pancreatic carcinoma, or neuroendocrine tumors) were excluded. Other exclusion criteria were (1) studies reporting results which included mixed pathological entities [i.e., mixed hepato-cholangiocarcinoma, mixed adeno-neuroendocrine carcinomas (MANEC)]; (2) publications in which techniques other than IHC and ISH were employed (with no data for IHC or ISH available); and (3) studies in which HER2 pathway analysis was performed following successful anti-HER2 therapy were excluded due to patient selection bias. When studies reporting the same series of patients were identified (“duplicate data”), the study with the greater number of informative patients for the primary end point of this review was selected for inclusion.

### Search strategy

A systematic search was conducted utilizing the PubMed/MEDLINE electronic data base (updated 20 November 2015); no dates of publication or language limits were applied. The following two search strategies were employed:

1. her2[All Fields] AND ((“cholangiocarcinoma”[MeSH Terms] OR “cholangiocarcinoma”[All Fields]) OR ((“biliary tract”[MeSH Terms] OR (“biliary”[All Fields] AND “tract”[All Fields]) OR “biliary tract”[All Fields]) AND (“carcinoma”[MeSH Terms] OR “carcinoma”[All Fields])) OR ((“gallbladder”[MeSH Terms] OR “gallbladder”[All Fields]) AND (“carcinoma”[MeSH Terms] OR “carcinoma”[All Fields])) OR (ampullary[All Fields] AND (“carcinoma”[MeSH Terms] OR “carcinoma”[All Fields])) OR ((“carcinoma”[MeSH Terms] OR “carcinoma”[All Fields]) AND (“ampulla of vater”[MeSH Terms] OR (“ampulla”[All Fields] AND “vater”[All Fields]) OR “ampulla of vater”[All Fields])));
2. her3[All Fields] AND ((“cholangiocarcinoma”[MeSH Terms] OR “cholangiocarcinoma”[All Fields]) OR ((“biliary tract”[MeSH Terms] OR (“biliary”[All Fields] AND “tract”[All Fields]) OR “biliary tract”[All Fields]) AND (“carcinoma”[MeSH Terms] OR “carcinoma”[All Fields])) OR ((“gallbladder”[MeSH Terms] OR “gallbladder”[All Fields]) AND (“carcinoma”[MeSH Terms] OR “carcinoma”[All Fields])) OR (ampullary[All Fields] AND (“carcinoma”[MeSH Terms] OR “carcinoma”[All Fields])) OR ((“carcinoma”[MeSH Terms] OR “carcinoma”[All Fields]) AND (“ampulla of vater”[MeSH Terms] OR (“ampulla”[All Fields] AND “vater”[All Fields]) OR “ampulla of vater”[All Fields]))) OR ((“receptor, erbb-2”[MeSH Terms] OR “genes, erbb-2”[MeSH Terms]) AND (((“cholangiocarcinoma”[MeSH Terms] OR “cholangiocarcinoma”[All Fields]) OR ((“biliary tract”[MeSH Terms] OR (“biliary”[All Fields] AND “tract”[All Fields]) OR “biliary tract”[All Fields]) AND (“carcinoma”[MeSH Terms] OR “carcinoma”[All Fields])) OR ((“gallbladder”[MeSH Terms] OR “gallbladder”[All Fields]) AND (“carcinoma”[MeSH Terms] OR “carcinoma”[All Fields])) OR (ampullary[All Fields] AND (“carcinoma”[MeSH Terms] OR “carcinoma”[All Fields]))) OR ((“carcinoma”[MeSH Terms] OR “carcinoma”[All Fields]) AND (“ampulla of vater”[MeSH Terms] OR (“ampulla”[All Fields] AND “vater”[All Fields]) OR “ampulla of vater”[All Fields])))).

Meeting abstracts from the American Society of Clinical Oncology (ASCO), European Society of Medical Oncology Congress (ESMO), and American Association for Cancer Research (AACR), presented over the last 5 years (2010–2015), were also reviewed using the following keywords: “her2” OR “her3” AND (“cholangiocarcinoma,” “biliary tract carcinoma,” “gallbladder carcinoma,” “ampullary carcinoma,” “carcinoma of ampulla of vater”).

Reference lists of eligible studies were cross-checked manually to identify potentially eligible articles.

### Primary and secondary objectives

The primary objective of this systematic review and meta-analysis was to assess the prevalence of HER2 overexpression (measured by IHC) in patients with BTC, with the primary end point being mean HER2 expression rate.

Secondary objectives included HER2 amplification (measured by ISH) both in the whole population (“unselected population”) and in the population of patients with HER2 overexpression by IHC (“selected population”) and HER3 overexpression (measured by IHC) and amplification (measured by ISH). HER2 and HER3 expression and amplification were analyzed by primary tumor site; HER2 expression was also analyzed by quality of expression assessment (“high quality” vs. “low quality”) and by region (Western vs. Asian). Correlation between HER2 and HER3 expression and between HER2 expression and HER2 amplification (in “unselected population”) was also assessed.

### Data collection

Eligibility for each of the studies was assessed by one of the authors (SG); queries were discussed with a second author (AL). Same process was followed for data collection. The total number of patients in each study together with numerator and denominator for each one of the reported rates were collected.

In order to perform the planned subgroup analyses, the following additional data were extracted from manuscripts (if available): primary site (CC, IHCC, EHCC, GBC, or AC) and ethnicity/region of patients involved in the study (Western vs. Asian). Tumor site was also subdivided into extrahepatic BTCs (EH-BTCs) which include EHCC, GBC, and AC and IHCC. In addition, eligible studies were classified according to the quality of HER expression assessment: studies were considered to be “high quality” (HQ) when moderate/strong HER2/HER3 overexpression was used to classify tumors, whereas studies were classified as “low quality” (LQ) when the HER2/HER3 overexpression threshold was not specified and/or not reported by authors or when “any” HER2/HER3 expression (including IHC 1+) was used.

For assessment of HER2 amplification rate, studies were classified according to the population in which ISH was performed: “unselected population” referred to studies in which the ISH was performed in the whole study population regardless of IHC results, while the term “selected population” was employed for those studies in which ISH was performed only in patients with overexpression of HER2 according to IHC.

### Statistical analysis

The Stata/MP v.12 package was used for the statistical analysis. Mean and 95 % confidence intervals (95 % CI) were calculated for reported HER2/HER3 expression/amplification rate. Mean HER2/HER3 expression/amplification rates were calculated for each one of the prespecified subgroup analyses: primary tumor site, region, quality of HER2 expression assessment, and population in which ISH was performed. All these analyses were repeated for each one of the tumor sites. Shapiro-Wilks normality test was performed for continuous variables; based on these results, parametric/nonparametric tests were used for the statistical analysis of the results and comparison of expression/amplification rates between subgroups employing Student’s *t* test or Wilcoxon rank-sum test as appropriate. Correlation was assessed using Pearson or Spearman’s rho as appropriate, according to whether variables followed a normal distribution or not.

## Results

### Eligible studies

**Fig. 2:**
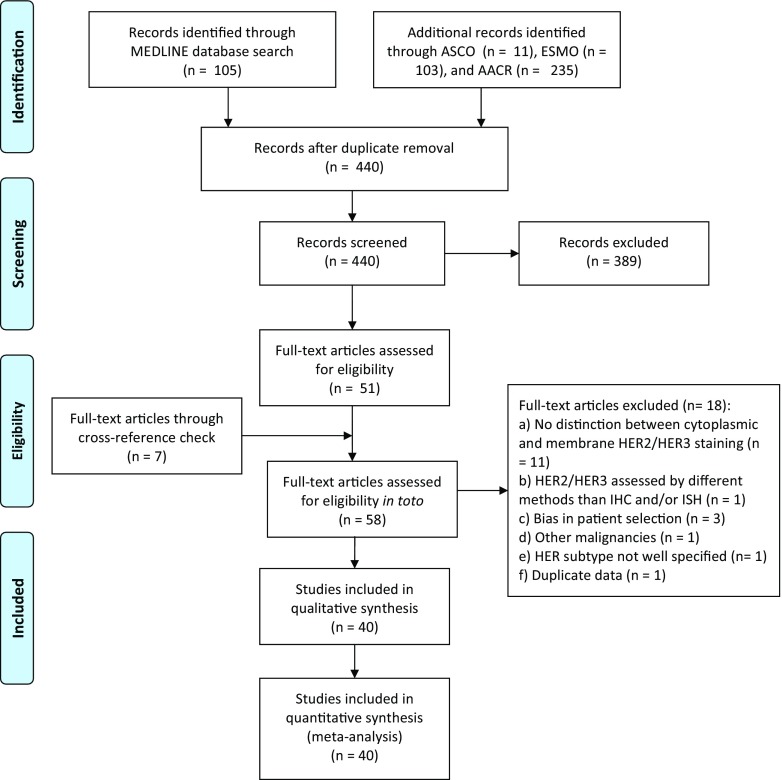
PRISMA flow diagram

Figure 2 summarizes the PRISMA flow diagram for selection of eligible studies [28]; a total of 454 results were obtained from the searches in PubMed/MEDLINE (*n* = 105), ASCO (*n* = 11), ESMO (*n* = 103), and AACR (*n* = 235). Of these, 14 were duplicates and 389 did not meet the inclusion criteria and were therefore excluded. Of the 51 studies which appeared to be eligible after the initial screen, a full-text search was carried out. In addition, seven full-text records through cross-reference checking were identified for a total of 58 studies assessable for eligibility. Eighteen studies were excluded after the full-text review as per our inclusion/exclusion criteria: 11 studies did not report an optimal distinction between cytoplasmic and membrane HER2/HER3 staining [29–39]; one study employed a method other than IHC and/or ISH for evaluating HER2/HER3 expression with no IHC/ISH data reported [40]; three studies reported HER2 analysis following successful targeting therapy and were therefore excluded due to selection bias [10, 41, 42]; one study reported joined results for BTCs and pancreatic cancer with no specific data for BTC patients [43]; one study did not report which member of the HER family was being assessed [44]; and one study reported “duplicate data” [45].

### Patient population

Forty studies were included in the final analysis, reporting a total of 3839 patients with a diagnosis of BTC [46–85] (Table 2). All studies were retrospective series, with a median number of 53 patients per study (range 6–804).

**Table 2:** HER2 and/or HER3 expression by immunohistochemistry (IHC) and/or amplification by *in situ* hybridization (ISH) in biliary tract carcinomas

Study	Country	*N*	Primary	HER2/IHC		HER2/ISH		HER3/IHC		HER3/ISH	
				*N*	%	*N*	%	*N*	%	*N*	%
Brunt EM	USA (Western)	6	CC	4/6	66.7	NR		NR		NR	
Collier JD 1992	UK (Western)	10	CC	0/10	0	NR		NR		NR	
Lei S 1995	USA (Western)	6	AC	2/6	33.3	NR		NR		NR	
Chow NH 1995	Taiwan (China) (Asian)	18	IHCC	5/18	27.8	NR		NR		NR	
		18	AC	5/18	27.8	NR		NR		NR	
		11	GBC	7/11	63.6	NR		NR		NR	
Vaidya P 1996	Japan (Asian)	14	EHCC	10/14	71.4	NR		4/14	28.6	NR	
		13	AC	9/13	69.2	NR		4/13	30.8	NR	
Terada T 1998	Japan (Asian)	47	CC	33/47	70	NR		NR		NR	
Kim YW 2001	Not specified	71	GBC	33/71	46.5	NR		NR		NR	
Ajiki T 2001	Japan (Asian)	30	AC	7/30	23	NR		NR		NR	
Ukita Y 2002	Japan (Asian)	22	IHCC	18/22	82	22/22	100	NR		NR	
Endo K 2002	Japan, Thailand, USA (n/a)	71	CC	21/71	29.6	NR		NR		NR	
Altimari A 2003	Italy (Western)	48	IHCC	2/48	4	2/48	4	NR		NR	
Matsuyama S 2004	Japan (Asian)	43	GBC	4/43	9.4	NR		NR		NR	
KIM HJ 2005	South Korea (Asian)	20	CC	5/20	25	NR		NR		NR	
Nakazawa K 2005	Japan (Asian)	28	IHCC	0/28	0	NR		NR		NR	
		78	EHCC	4/78	5.1	NR		NR		NR	
		89	GBC	14/89	15.7	NR		NR		NR	
		26	AV	3/26	11.5	NR		NR		NR	
		71	BTC	–		15/71	21.1	–		–	
		19	BTC	–		15/19	79	–		–	
Settakorn J 2005	Australia/Thailand (n/a)	31	IHCC	10/31	32.3	NR		NR		NR	
Ogo Y 2006	Japan (Asian)	72	BTCs	47/72	65	NR		NR		NR	
Kim JH 2007	South Korea (Asian)	55	EHCC	16/55	29.1	10/55	18.1	NR		NR	
Kawamoto T 2007	USA/Chile (Western)	21	IHCC	7/21	33.3	0/14	0	NR		NR	
		16	EHCC	5/16	33.3	3/14	21.4	NR		NR	
		77	GBC	24/77	31.2	14/67	20.9	NR		NR	
Yoshikawa D 2008	Japan (Asian)	106	IHCC	1/106	0.9	NR		NR		NR	
		130	EHCC	11/130	8.5	NR		NR		NR	
Miyahara n 2008	Japan (Asian)	51	GBC	16/51	31	4/16	25	NR		NR	
Joo HH 2007	South Korea (Asian)	112	BTCs	5/112	4.5	NR		NR		NR	
Puhalla H 2007	Austria (Western)	55	GBC	7/55	13	NR		NR		NR	
Kaufmann M 2008	USA (Western)	16	GBC	1/16	6.3	NR		NR		NR	
Baumhoer D 2008	Switzerland, Germany, Italy (Western)	82	AV	NR		5/82	6	NR		NR	
Choi HJ 2009	Not specified	50	IHCC	36/50	72	NR		NR		NR	
Aloysius MM 2009	UK (Western)	29	EHCC	0/29	0	NR		NR		NR	
		22	AV	0/22	0	NR		NR		NR	
Harder J 2009	Germany (Western)	124	BTCs	25/124	20.2	6/25	24	NR		NR	
Shafizadeh N 2010	USA (Western)	26	IHCC	0/26	0	NR		NR		NR	
		19	EHCC	2/19	10.5	NR		NR		NR	
		6	GBC	0/6	0	NR		NR		NR	
Pignochino Y 2010	Italy (Western)	17	IHCC	0/10	0	NR		NR		NR	
		19	EHCC	4/19	21	2/4	50	NR		NR	
		13	GBC	1/10	10	1/1	100	NR		NR	
Toledo C 2012	Chile (Western)	12	GBC	4/12	33	0/12	0	NR		NR	
Kumari N 2012	India (Asian)	104	GBC	14/104	13.4	NR		NR		NR	
Lee HJ 2012	South Korea (Asian)	230	EHCC	13/224	6	NR		90/230	39	NR	
Roa Iván 2013	Chile (Western)	187	GBC	62/187	31.11	NR		NR		NR	
Wang W 2014	China (Asian)	58	IHCC	0/90	0	0/90	0	NR		NR	
		94	EHCC	4/90	4.4	3/94	3.5	NR		NR	
Graham RP 2014	USA (Western)	100	BTCs	3/100	3	3/3	100	NR		NR	
Yang X 2014	China (Asian)	65	IHCC	0/65	0	0/65	0	8/65	12.3	NR	
		110	EHCC	5/110	4.5	8/108	7.4	13/110	11.8	NR	
Kawamoto T 2015	USA	47	GBC	15/47	32	8/47	17	16/47	34	12/47	26
	Japan (n/a)	66	CC	15/66	23	15/66	23	19/66	29	18/66	27
Hechtman J 2015	USA (Western)	106	AC	27/106	25.5	13/100	13	NR		NR	
Oliveira Fernandes VT 2015	Brazil (Western)	38	CC	11/38	30	NR		NR		NR	
Holcombe RF 2015	USA (Western)	126	EHCC	NR		NR	18	NR		NR	
		434	IHCC	NR		NR	1.5	NR		NR	
		244	GBC	NR		NR	15	NR		NR	

*CC* cholangiocarcinoma, *IHCC* intrahepatic cholangiocarcinoma *EHCC* extrahepatic cholangiocarcinoma, *GBC* gallbladder carcinoma, *AC* carcinoma of ampulla of Vater, *BTCs* biliary tract carcinomas, *NR* not reported, *n/a* not applicable

According to the primary tumor site, the number of studies and number of patients reported were as follows: CC (24 studies; 2102 patients; 55 % of all patients reported), IHCCs (13 studies; 924 patients; 24 % of all patients reported; 44 % of all CC patients), EHCCs (12 studies; 920 patients; 24 % of all patients reported; 44 % of all CC patients), GBCs (15 studies; 1026 patients; 27 % of all patients reported), and ACs (8 studies; 303 patients; 8 % of all patients reported). In seven studies (258 patients; 7 % of all patients reported; 12 % of all CC patients), the type of CC (IHCC vs. EHCC) was not specified. In four studies (408 patients; 10 % of all patients reported), the type of BTC was not specified. Eighteen out of 40 (45 %) studies were conducted in Western countries and 17 (43 %) in Asian population, while the remaining 5 (12 %) studies were mixed or not specified (Table 2).

### HER2 expression (IHC)

Thirty-eight studies reported HER2 positivity assessed by IHC (Table 3); two studies did not perform HER2 IHC analysis [66, 85]. Technical details regarding this assessment were available for 37 of the 38 studies: in the remaining study, this data was not available [71]. The most commonly used (23 of 37 studies; 62 %) anti-HER2 antibody was polyclonal (Dako®, Dakopatts®, Nichirei®, or Zymed Lab®), followed by monoclonal antibody in 13 studies (35 %) (Triton Biosciences Inc.®, Immunotech®, DAKO®, Oncogene®, Zymed Lab®, Carpinteria®, Ventana®, or Novocastra®); this information was not available in one study (3 %). HER2 expression was qualitatively analyzed in 5 out of 37 (14 %) studies, while a semiquantitative score, estimating the fraction of positive cells, was used in 32 studies (86 %) (Table 3).

**Table 3:** Descriptive features of immunohistochemistry (IHC) and *in situ* hybridization (ISH) for HER2 and HER3 in biliary tract carcinomas

Study	HER2						HER3				
	Platform	IHC scoring	Qualitative/semiquantitative	LQ/HQ assessment	ISH	Selection of patients for ISH	Platform	IHC scoring	Qualitative/semiquantitative	LQ/HQ assessment	ISH
Brunt EM 1992	MAb (Triton Biosciences Inc., USA)	Weak (1+), moderate (2+) or strong (3+), and focal or diffuse	Semiquantitative	LQ	n/a	n/a	n/a	n/a	n/a	n/a	n/a
Collier JD 1992	MAb NCL-CB11	Negative and positive	Qualitative	LQ	n/a	n/a	n/a	n/a	n/a	n/a	n/a
Lei S 1995	MAb CB11	Negative, weak, moderate, or strong	Semiquantitative	HQ	n/a	n/a	n/a	n/a	n/a	n/a	n/a
Chow NH 1995	MAb-1 (Triton Biosciences Inc., USA)	Focal staining (+) and diffuse staining (++)	Qualitative	LQ	n/a	n/a	n/a	n/a	n/a	n/a	n/a
Vaidya P 1996	PolyAb (Dakopatts, Denmark)	A 4-point scale: 0, 1+, 2+, and 3+	Semiquantitative	HQ	n/a	n/a	Novocastra Lab. Ltd., UK	A 4r-point scale: 0, 1+, 2+, and 3+	Semiquantitative	HQ	n/a
Terada T 1998	MAb 3B5 (Immunotech, France)	A 5-point scale: –, +, ++, +++, and ++++	Semiquantitative	LQ	n/a	n/a	n/a	n/a	n/a	n/a	n/a
Kim YW 2001	PolyAb (Dako, USA)	A 4-point scale: 0, 1+, 2+, and 3+	Semiquantitative	LQ	n/a	n/a	n/a	n/a	n/a	n/a	n/a
Ajiki T 2001	PolyAb (Dako, Denmark)	Immunoreactivity present in more than 10 % of tumor cells	Semiquantitative	LQ	n/a	n/a	n/a	n/a	n/a	n/a	n/a
Ukita Y 2002	MAb 3B5 (Immunotech, France)	A 5-point scale: –, +, ++, +++, and ++++	Semiquantitative	LQ	FISH	Unselected	n/a	n/a	n/a	n/a	n/a
Endo K 2002	Mab F-11 (Dako, USA)	A 5-point scale: 0, ±, +, 2+, and 3+	Semiquantitative	HQ	n/a	n/a	n/a	n/a	n/a	n/a	n/a
Altimari A 2003	PolyAb HercepTest (Dako, Denmark)	A 4-point scale: 0, 1+, 2+, and 3+	Semiquantitative	HQ	CISH	Unselected	n/a	n/a	n/a	n/a	n/a
Matsuyama S 2004	PolyAb HercepTest (Dako, Denmark)	A 4-point scale: 0, 1+, 2+, and 3+	Semiquantitative	HQ	n/a	n/a	n/a	n/a	n/a	n/a	n/a
Kim HJ 2005	MAb (Oncogene, USA)	Negative or positive	Qualitative	LQ	n/a	n/a	n/a	n/a	n/a	n/a	n/a
Nakazawa 2005	PolyAb (Nichirei, Japan)	A 4-point scale: 0, 1+, 2+, and 3+	Semiquantitative	HQ	FISH	Unselected and selected	n/a	n/a	n/a	n/a	n/a
Settakorn J 2005	PolyAb (HercepTest, Dako)	A 4-point scale: 0, 1+, 2+, and 3+	Semiquantitative	HQ	n/a	n/a	n/a	n/a	n/a	n/a	n/a
Ogo Y 2006	PolyAb (HercepTest Dako, A0485)	A 4-point scale: 0, 1+, 2+, and 3+	Semiquantitative	HQ	n/a	n/a	n/a	n/a	n/a	n/a	n/a
Kim HJ 2007	PolyAb (Zymed Lab, USA)	A 4-point scale: 0, 1+, 2+, and 3+	Semiquantitative	HQ	CISH	Unselected	n/a	n/a	n/a	n/a	n/a
Kawamoto T 2007	PolyAb HercepTest (Dako, Denmark)	A 4-point scale: 0, 1+, 2+, and 3+	Semiquantitative	HQ	FISH	Unselected	n/a	n/a	n/a	n/a	n/a
Yoshikawa D 2008	Polyab HercepTest (Dako, Denmark)	A 4-point scale: 0, 1+, 2+, and 3+	Semiquantitative	HQ	n/a	n/a	n/a	n/a	n/a	n/a	n/a
Baumhoer D 2008	n/a	n/a	n/a	LQ	FISH	Unselected	n/a	n/a	n/a	n/a	n/a
Miyahara N 2008	PolyAb HercepTest (Dako)	A 4-point scale: 0, 1+, 2+, and 3+	Semiquantitative	HQ	FISH	Selected	n/a	n/a	n/a	n/a	n/a
Joo HH 2007	PolyAb (Zymed)	A distinctive membrane staining was referred as positive	Qualitative	LQ	n/a	n/a	n/a	n/a	n/a	n/a	n/a
Puhalla H 2007	PolyAb HercepTest (Dako)	A 4-point scale: 0, 1+, 2+, and 3+	Semiquantitative	HQ	n/a	n/a	n/a	n/a	n/a	n/a	n/a
Kaufmann M 2008	MAb CB11 (Carpinteria, USA)	A 4-point scale: 0, 1+, 2+, and 3+	Semiquantitative	HQ	n/a	n/a	n/a	n/a	n/a	n/a	n/a
Aloysius MM 2009	PolyAb HercepTest (Dako)	A 4-point scale: 0, 1+, 2+, and 3+	Semiquantitative	HQ	n/a	n/a	n/a	n/a	n/a	n/a	n/a
Harder J 2009	PolyAb (Dako REAL™, Denmark)	A 4-point scale: 0, 1+, 2+, and 3+	Semiquantitative	HQ	FISH	Selected	n/a	n/a	n/a	n/a	n/a
Choi 2009	NR	NR	NR	LQ	n/a	n/a	n/a	n/a	n/a	n/a	n/a
Shafizadeh N 2010	MAb CB11, (Ventana, USA)	A 4-point scale: 0, 1+, 2+, and 3+	Semiquantitative	HQ	n/a	n/a	n/a	n/a	n/a	n/a	n/a
Pignochino Y 2010	PolyAb HercepTest (Dako)	A 4-point scale: 0, 1+, 2+, and 3+	Semiquantitative	HQ	FISH	Selected	n/a	n/a	n/a	n/a	n/a
Toledo C 2012	MAb NCL-CBE-356 (Novocastra)	Positive or negative	Qualitative	LQ	FISH	Unselected	n/a	n/a	n/a	n/a	n/a
Kumari N 2012	PolyAb (Dako)	A 4-point scale: 0, 1+, 2+, and 3+	Semiquantitative	HQ	n/a	n/a	n/a	n/a	n/a	n/a	n/a
Lee HJ 2012	PolyAb A0485 (Dako, Denmark)	A 4-point scale: 0, 1+, 2+, and 3+	Semiquantitative	HQ	n/a	n/a	MAb RTJ.2 (Santa Cruz, USA)	Staining intensity and percentage of positive cells^a^	Semiquantitative	HQ	n/a
Roa I 2013	MAb NCL-CB11 (Novocastra)	A 4-point scale: 0, 1+, 2+, and 3+	Semiquantitative	HQ	n/a	n/a	n/a	n/a	n/a	n/a	n/a
Wang W 2014	PolyAb (Dako, USA)	A 4-point scale: 0, 1+, 2+, and 3+	Semiquantitative	HQ	FISH	Unselected	n/a	n/a	n/a	n/a	n/a
Graham 2014	PolyAb HercepTest (Dako, USA)	A 4-point scale: 0, 1+, 2+, and 3+	Semiquantitative	HQ	FISH	Selected	n/a	n/a	n/a	n/a	n/a
Yang X 2014	PolyAb (Dako)	A 4-point scale: 0, 1+, 2+, and 3+	Semiquantitative	HQ	FISH	Unselected	sc-415 (Santa Cruz Biotechnology, USA)	Rajikumar score^b^	Semiquantitative	HQ	n/a
Kawamoto T 2015	PolyAb HercepTest II (Dako A/S, Denmark)	A 4-point scale: 0, 1+, 2+, and 3+	Semiquantitative	HQ	FISH	Unselected	Spring Bioscience, USA	A 4-point scale: 0, 1+, 2+, and 3+	Semiquantitative	HQ	FISH
Hechtman J 2015	MAb 4B5 (Ventana Medical Systems, USA)	A 4-point scale: 0, 1+, 2+, and 3+	Semiquantitative	HQ	CISH	Unselected	n/a	n/a	n/a	n/a	n/a
Oliveira Fernandes VT 2015	NR	A 4-point scale: 0, 1+, 2+, and 3+	Semiquantitative	HQ	n/a	n/a	n/a	n/a	n/a	n/a	n/a
Holcombe RF 2015	n/a	n/a	n/a	LQ	FISH	Unselected	n/a	n/a	n/a	n/a	n/a

*NR* not reported, *n/a* not applicable, *LQ* low quality, *HQ* high quality, *FISH* fluorescence *in situ* hybridization, *CISH* chromogenic *in situ* hybridization

^a^HER3 was scored based on the intensity of staining as 0 (negative), 1 (weak), or 2 (strong) and the percentage of positive epithelial cells as 0 (<5 %), 1 (6–25 %), 2 (26–50 %), 3 (51–75 %), or 4 (>76 %). A Histoscore was generated as the product of intensity and area. The Histoscore was then dichotomized into no/lower expression (Histoscore, 0–6) and overexpression (Histoscore, 8)

^b^Rajikumar score based on two parameters: staining intensity (range, 0–3) and percentage of positive cells [range, 0–4 = 0 (0–10 %); 1 (11–25 %), 2 (26–50 %), 3 (51–75 %), 4 (>76 %)]. Slides with scores of ≥8 were classified as overexpression and slides with scores <8 as nonoverexpression

Globally, the mean HER2 expression rate was 26.5 % (95 % CI, 18.9–34.1 %; Table 4). There were no statistically significant differences between regions (Asian mean HER2 expression rate 28.4 % (95 % CI 14.5–42.3 %) vs. Western 19.7 % (95 % CI 10.1–29.2 %); *p* value 0.4936; Table 4). With respect to the quality of HER2 expression assessment, LQ studies (11 studies; 27 % of all studies reporting HER2-IHC data) had a significantly higher mean HER2 expression rate compared to HQ studies (27 studies; 68 % of all studies reporting HER2-IHC data): 41.7 % (95 % CI 22.9–60.5 %) vs. 20.3 % (95 % CI 13.3–27.4 %), respectively, *p* value 0.0336; Table 4.

**Table 4:** HER2 expression and amplification results in biliary tract carcinomas

HER2 status		No. of studies	Expression rate mean (95 % CI, %)	*p* value
Overall expression by IHC	All	38	26.5 % (18.9–34.1 %)	
By ethnicity	Asian	17	28.4 % (14.5–42.3 %)	Ref
	Western	16	19.7 % (10.1–29.2 %)	0.4936
By IHC assessment (quality)	Low quality (LQ)	11	41.7 % (22.9–60.5 %)	Ref
	High quality (HQ)	27	20.3 % (13.2–27.5 %)	***0.0336***
By site of primary (HQ studies only)	IHCC	8	4.8 % (0–14.5 %)	Ref
	EH-BTC	28	19.9 % (12.8–27.1 %)	***0.0049***
	EHCC	11	17.4 % (3.4–31.4 %)	***0.0134***
	GBC	12	19.1 % (11.2–26.8 %)	***0.0123***
	AC	5	27.9 % (0–60.7 %)	0.0642
Overall amplification by ISH	All	16	30.1 % (11.7–48.5 %)	
By site of primary	IHCC	6	17.6 % (0–60.1 %)	Ref
	EH-BTC	14	22.5 % (7.9 %–37.2 %)	***0.0468***
By patient selection	Unselected	12	17.9 % (0.1–35.4 %)	Ref
	Selected	5	57.6 % (16.2–99 %)	***0.0072***

*LQ* low quality, *HQ* high quality, *ISH in situ* hybridization, *IHCC* intrahepatic cholangiocarcinoma, *EHCC* extrahepatic cholangiocarcinoma, *EH-BTCs* extrahepatic biliary tract cancers, *GBC* gallbladder carcinoma, *AC* ampulla of Vater carcinoma, *Ref* category used as reference for comparisons

Bold-italics represent statistically significant results

In all 38 studies, no differences in HER2/IHC expression rates were found between tumor sites when considering all studies, regardless of the quality of HER2 expression assessment (Table 4). In contrast, when only HQ studies were considered, the mean HER2 overexpression rate in EH-BTCs was statistically significantly higher to IHCCs (Table 4). Moreover, mean HER2 overexpression rate was statistically significantly higher in EHCCs compared to IHCCs and in GBCs compared to IHCCs, whereas there was only a marginal difference between ACs and IHCCs (Table 4).

### HER2 amplification (ISH)

HER2 amplification analysis was performed in 16 studies: applying FISH and CISH in 13 (81 %) and 3 (19 %) studies, respectively (Table 3). Mean HER2 amplification rate was 30.1 % (95 % CI 11.7–48.5 %) when all BTCs were analyzed together (Table 4). When all studies were included (regardless of applying ISH for “selected” or “unselected” population), mean HER2 amplification rate was statistically significantly higher in patients with EH-BTCs compared to IHCCs (Table 4). Interestingly, the mean HER2 amplification rate was higher in the five studies [59, 68, 72, 73, 78] in which ISH test was performed in “selected” population when compared to the 12 studies in which ISH test was applied to “unselected population” only [17.9 % (95 % CI 0.1–35.4 %) vs. 57.6 % (95 % CI 16.2–99 %), *p* value 0.0072] [55, 56, 59, 63, 64, 66, 77, 80–83, 85] (Table 4). Nakazawa et al. reported data from 221 patients, 71 of whom had FISH testing performed: meaningful differences in HER2 amplification rate were shown between “unselected” [15/71 (21 %)] and “selected” [15/19 (79 %)] populations [59] (Table 2).

### Correlation between HER2 expression and amplification

**Fig. 3:**
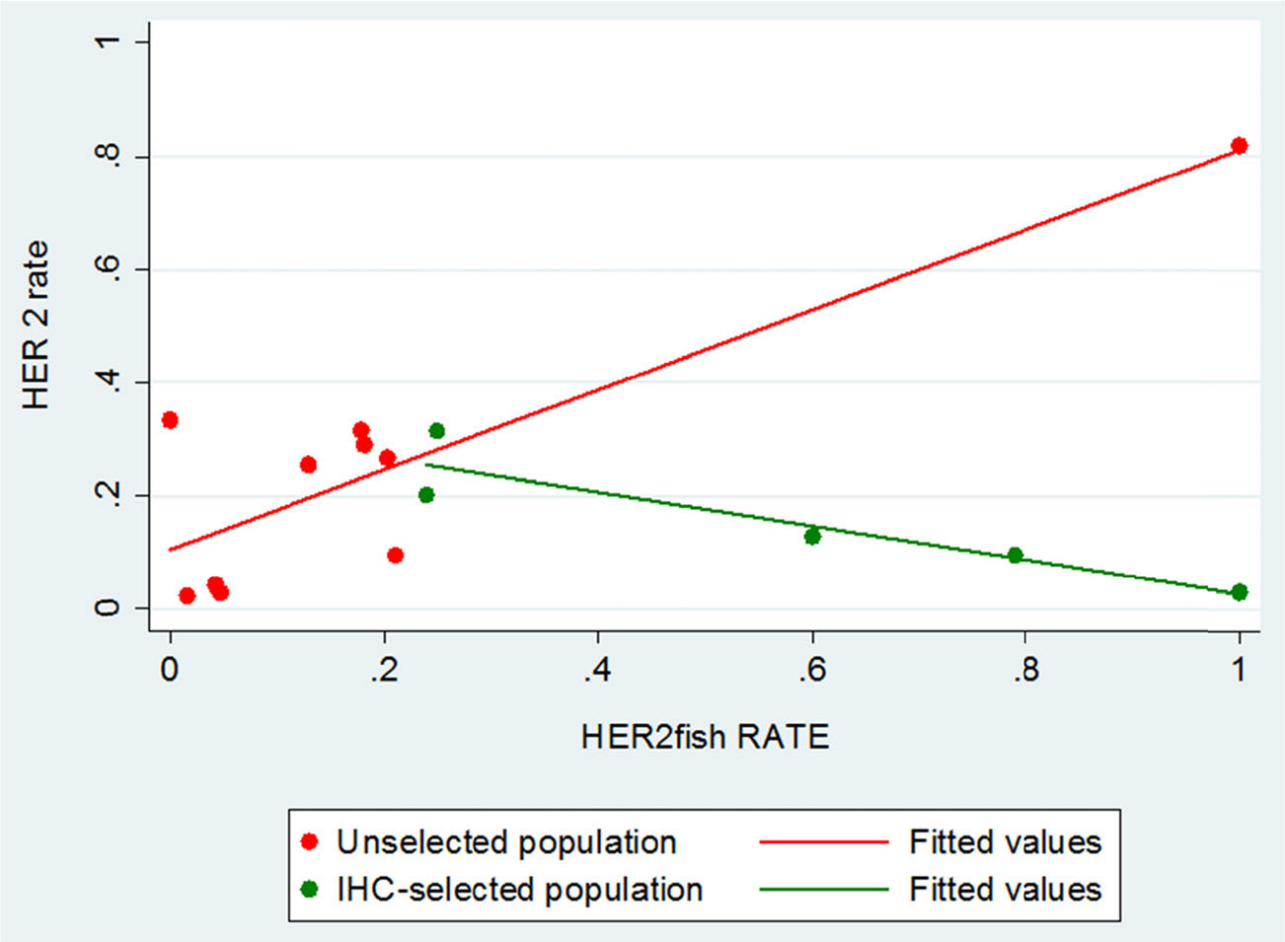
Correlation between HER2 expression and amplification

Ten studies [55, 56, 59, 63, 64, 77, 80, 82, 83, 85] and five studies [59, 68, 72, 73, 78] had data for both HER2 expression and amplification in the “unselected” and “selected” population, respectively. While no statistically significant correlation was observed in studies with the “selected” population (five studies; Spearman rho = −0.9; *p* value 0.037), a better correlation (although not statistically significant) was shown in “unselected” patients (10 studies; Spearman rho 0.38; *p* value 0.2763) (Fig. 3).

### HER3 expression and amplification

HER3 expression rate was reported in four studies in which different commercially available antibodies were used (Novocastra®, Santa Cruz®, or Spring Bioscience®). All four studies had a HQ HER3 expression assessment (Table 3). The pooled mean overall HER3 overexpression rate was 27.9 % (95 % CI 9.7–46.1 %) [51, 76, 81, 83]; only one study reported HER3 amplification rate (26.5 %) [83] (Table 2).

Further subgroup analyses for HER3 expression and amplification were not possible due to limited number of studies.

### Correlation between HER2 and HER3 overexpression

**Fig. 4:**
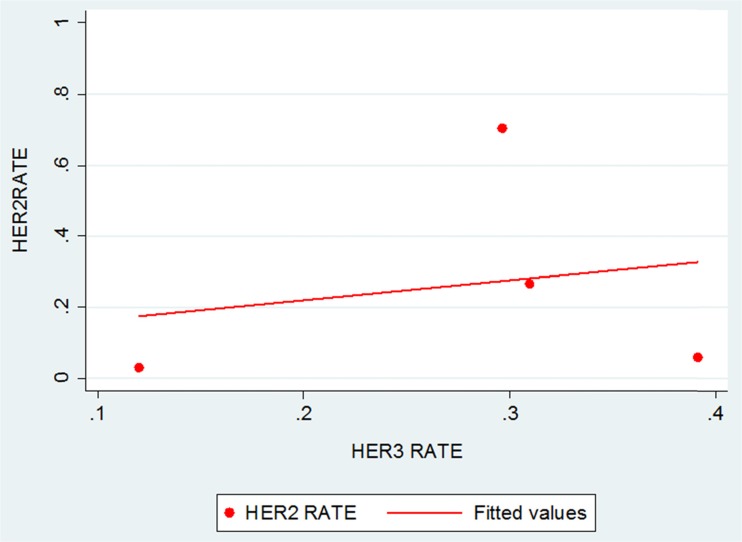
Correlation between HER2 and HER3 expression

All of the four studies with HER3 expression data had HER2 expression data available for correlation analysis [51, 76, 81, 83]. No statistically significant correlation was identified between HER2 and HER3 overexpression (four studies; Spearman rho = 0.2; *p* value 0.8) (Fig. 4).

## Discussion

The present systematic review and meta-analysis found that there is a higher moderate/strong HER2 expression rate (~20 %) in extrahepatic biliary tract carcinomas than in IHCC (<5 %). In a previous meta-analysis, Wiggers et al. also reported a statistically significant higher expression of HER2 in EHCC [risk ratio 0.22 (95 % CI, 0.07–0.65)] than in IHCC [86]. However, this work was based on a smaller number of studies (five) detecting HER2 expression in only IHCC and EHCC, excluding GBC and AC, and did not provide any information about ISH testing. Subgroup analysis by site of primary in the current study suggested that HER2 overexpression was higher in EHCC, GBC, and AC tumors than in IHCCs [of note, this difference was not statistically significant between the group of IHCC and ACs, probably due to a smaller sample size of this subgroup (303 patients)].

Interestingly, in the IHC “selected population” (patients with moderate/strong expression by IHC), HER2 amplification rate was found to be ~60 %. Therefore, these findings (moderate/strong HER2 expression rates in EH-BTCs and FISH rates in IHC “selected” patients) suggest around 10–20 % of EH-BTCs can be virtually considered HER2 upregulated. In a recent case series of 211 consecutive GBC tumors, 16.6 % of tumors were globally found to be HER2 positive when IHC3+ and IHC 2+/FISH-amplified tumors were considered altogether [87]. According to international HER2 assessment criteria used for breast and gastric cancer [6, 26, 27], it may be assumed that BTCs scoring 3+ on immunohistochemistry should be interpreted as positive, while the application of *in situ* hybridization (fluorescence or chromogenic) could be carried out only in tumors with an ambiguous (IHC 2+) score.

These results, in addition to some preclinical data demonstrating that constitutive overexpression of activated HER2 can result in cholangiocarcinoma development [88], provide some support that the HER2 protein may play an important role in extrahepatic biliary carcinogenesis. Consequently, the HER2 pathway may be considered as a potential actionable target in EH-BTCs. Inhibition of HER2-mediated signaling is an established therapeutic strategy in HER2-positive breast and gastric cancer in which HER2 overexpression rates (up to 20 %) are similar to that found in EH-BTCs [26, 89]. Anti-HER2 therapy options might include the antibodies trastuzumab, pertuzumab, or trastuzumab emtansine (T-DM1) or the small-molecule, orally active, TKI, lapatinib [6, 90, 91].

Beyond HER2, in BTC, other biomarkers might be involved in cancer pathogenesis, prognosis, and resistance to therapy. In the current meta-analysis, approximately one in four patients had moderate/strong expression of HER3 or HER3 gene amplification. Most interestingly, HER2/HER3 co-expression in BTCs ranges from 9 to 53 % [76, 83] and has been demonstrated to be frequently associated with phosphorylation (activation) of HER2 and AKT [83]. HER3 is often correlated with poorly differentiated biliary tumors [81] and appears to be a poor prognostic factor in EHCCs [76], whereas the prognostic meaning of HER2 has not been completely clarified [79, 87]*.* Interestingly, the combination of pertuzumab and trastuzumab has been reported to induce a synergistic inhibition of *in vivo* tumor growth in BTCs, likely because of a more comprehensive blockade of HER2/HER3 signaling [83]. Moreover, HER4 was found to be overexpressed in 63.1 % of IHCCs and in 56.4 % of EHCCs, respectively, demonstrating to be a significant poor prognostic factor in EGFR-negative IHCC cases [81]. KRAS/NRAS mutations occur in 6.1–6.5 % of BTCs [73, 82, 85] and they appear to be mutually exclusive with HER2 amplification, at least in ACs [82]. Less frequently, BTCs harbor BRAF mutations (0–8.1 %) or PI3K mutations (7.3–10.2 %) [73, 82], while MET expression measured by IHC ranges from 5.6 to 44.1 % [54, 59, 62]. Importantly, investigational research should mainly define magnitude and prognostic impact of these biomarkers in BTCs and their correlation with HER2/HER3 pathway.

Therefore, due to inherent anatomical and molecular features, BTCs should no longer be classified as a singular entity and, in the future, differences in tumor location or tumor biology as well as an accurate distinction from other neoplastic entities should be carefully considered so as to minimize disappointing results in both clinical practice and scientific research.

This systematic review and meta-analysis has limitations, mainly linked to inter-study heterogeneity. In several studies, a clear definition of the primary tumor site was not available or results were not reported separately for each subgroup, thus limiting the eligible data for inclusion in subgroup analyses. Since no standardized techniques and scores to assess HER2 amplification and expression are available in BTCs, and because there are no internationally accepted and validated methods for HER3 testing established in any tumor, inconsistency in methodology may be an issue. Furthermore, the articles included in this meta-analysis covered a long period of time (1992 to 2015), and thus various laboratory assays were likely utilized to determine HER2 protein expression and gene amplification with different cutoff values for positivity employed. Differences in methodology, disease stage (early vs. advanced), tumor specimen (resection specimen vs. biopsy), site of tumor specimen (primary vs. metastases), IHC scoring system (qualitative vs. semiquantitative), threshold definition of IHC overexpression (provided vs. not), and/or choice of tumors in which the ISH test was applied (HER2 overexpression vs. no overexpression) may explain the wide range of both HER2 expression (0 to 82 %) and amplification (0 to 100 %) positivity reported in this review. Moreover, available literature indicates a certain variability between polyclonal and monoclonal antibodies in the ability to detect membranous HER2 protein, a higher level of concordance between IHC and ISH for polyclonal antibodies, and the possibility of influencing antigen retrieval through utilization of various application methods on tissue samples [92]. Due to the characteristics of the data reported, it was not possible to perform analysis of co-expression rate between HER2 and HER3 or concordance between IHC and ISH. Finally, no survival data was available, making it impossible to assess the prognostic implications of HER2/HER3 expression/amplification.

Despite the abovementioned limitations, this meta-analysis is the first study to systematically estimate the prevalence of HER2 and HER3 in all BTCs. Approximately one fifth of EH-BTCs are HER2 overexpressed, suggesting that the development of strategies against this receptor could be a reasonable therapeutic approach. Further data is required regarding the impact of co-expression of both HER2 and HER3. Standardization of ISH and IHC techniques, validation of scoring criteria for HER2 and HER3 immunohistochemistry, and assessment of concordance between IHC and ISH, focusing on the high intra-tumoral heterogeneity of HER2 membranous protein [87], are needed if the techniques are to be adopted to clinical practice. Assuming that an overexpression of HER2 of 5 % or less could be considered “un-interesting,” in this era of personalized medicine and spending review, our data may be particularly pertinent for the most cost-effective selection of patients with BTCs who may benefit from anti-HER2-targeted therapy.

Well-designed prospective clinical trials, for patients rigorously selected by HER2-positive tumors and, possibly, stratified by tumor location, are warranted to confirm the benefit of adding anti-HER2-targeted agents to chemotherapy in advanced disease. Given the lack of benefit reported for lapatinib in previous phase II trials in BTCs [93, 94] as well as in phase III trials in HER2-positive advanced gastric cancer [95, 96], alternative anti-HER2 therapies such as monoclonal antibodies trastuzumab and pertuzumab seem to be more promising.
